# Investigating mood-modification, withdrawal, and sensitization in compulsive sexual behaviour

**DOI:** 10.3389/fpsyt.2024.1421028

**Published:** 2024-10-11

**Authors:** Eli Sassover, Talma Kushnir, Aviv M. Weinstein

**Affiliations:** Psychology Department, Ariel University, Ariel, Israel

**Keywords:** compulsive sexual behaviour, sex addiction, tolerance, withdrawal, mood modifications

## Abstract

**Background and aims:**

Compulsive Sexual Behaviour (CSB), defined as a persistent failure to control repetitive sexual impulses, has been discussed as a pathological phenomenon for centuries. Various terms, such as excessive sexual behaviour, hyper-sexuality, compulsive sexual behaviour disorder (CSBD), or sexual addiction (SA), have been used to describe it, contributing to ongoing debates about its theoretical framework. The following three studies aim to empirically assess whether CSB exhibits key elements of behavioural addiction (mood-modification, sensitization/tolerance, and withdrawal).

**Method:**

Three studies, involving participants with and without CSB, were conducted. The mood-modification hypothesis was tested by exposing participants to short films inducing positive, negative, and emotionally neutral moods, followed by an evaluation of their craving for pornography. To test the sensitization hypothesis, participants viewed short films with varying levels of explicit sexual stimuli, and their level of actual wanting and liking were assessed through self-reports. For the withdrawal hypothesis, participants underwent a 10-day sexual abstinence, with self-reports of various symptoms, collected on pre-intervention, 3rd, 7th, and 10th days.

**Results:**

Contrary to previous studies of addiction, CSB participants didn’t show increased craving to mood induction and negative mood actually decreased craving for pornography. Secondly, they showed wanting to explicit sexual stimuli although it was not increased with explicitness. Finally, they demonstrated reduced withdrawal symptom during abstinence.

**Conclusion:**

The results of this study provide conflicting results concerning the model of behavioural addiction. There is supporting evidence for wanting in response to explicit pornography stimuli although it was not associated with increased explicitness. There is also evidence for reduced withdrawal during abstinence. Finally, there was no evidence that mood modification increases craving for pornography, negative mood actually decreased craving. Further research is needed to test the various models of CSB.

## Introduction

Compulsive Sexual Behaviour disorder CSBD is “a persistent pattern of failure to control intense, repetitive sexual impulses or urges resulting in repetitive sexual behaviour” (1). Excessive sexuality has been discussed as a pathological phenomenon since the first centuries of the first millennium (2). The characteristics of the disorder and its elements have been the subject of a great deal of debate in the past few decades. Different terms were assigned to describe this phenomenon. CSBD, excessive sexual behaviour, hyper-sexuality or sexual addiction (SA), are all different labels for the same phenomenon, reflecting different theoretical frameworks for understanding non-paraphilic undue sexual behaviour. The behaviour those concepts represent is maladaptive sexual behaviour that takes a lot of time daily and persists despite adverse consequences and despite efforts to stop it (3). The literature is still inconclusive regarding the prevalence and classification of CSBD. The main goal of this paper is to empirically examine three major elements of addiction and whether they are present in CSB, namely mood modification, withdrawal, and sensitization. We will first review the evolution of this classified disorder, then portray the core elements of addiction, followed by a literature review that associates CSB with these elements.

## Psychiatric model of CSBD

Orford (4) challenged the traditional views on sexual behaviour, arguing against labelling it as normal or exaggerated. Rather he emphasized the conflict between sexual desires and life demands. Carnes (5) introduced the concept of sexual addiction, sparking ongoing theoretical debates. Others, like Coleman (6), rejected the idea of viewing sex as a drug of choice and saw the hyper-sexuality as a compulsive reaction to an inner obsessive state. Given the enjoyable element of sex, Barth and Kinder (7) rejected the compulsive perspective and viewed hyper-sexuality as an expression of impulse control disorder.

## Current perspectives and ICD-11 classification

In 2010, Kafka (8) proposed the inclusion of hypersexual disorder as a distinct diagnosis in the upcoming Diagnostic and Statistical Manual of Mental Disorders (5th ed.; DSM-5; 9). When considering its categorical placement in the DSM-5, he suggested that the term “compulsive,” while suitable for describing certain features of the condition, deviates from the conventional DSM-based understanding of obsessive-compulsive spectrum disorders. Conversely, considering hypersexual disorder as a primary impulse disorder could potentially conflict with the current classification of other seemingly analogous biologically mediated appetitive behaviour disorders, such as bulimia nervosa (eating disorders) or hypersomnia (sleep disorders). In 2018 the ICD, in its 11th edition ([ICD-11]; 1) introduced the diagnosis of CSBD as “a persistent pattern of failure to control intense, repetitive sexual impulses or urges resulting in repetitive sexual behaviour”. Yet, this conceptualization didn’t put to rest the debates and different standpoints that are still being held, the addiction model is still being advocated, and the role of impulsivity over compulsivity is still being discussed (10–13). While compulsivity, impulsivity, and addiction, share some features, they differ in profound others. Addiction is characterized by withdrawal and tolerance phenomena that are not characterized by impulsivity and compulsivity (9).

## Current findings regarding CSBD and the aim of the current paper

Black et al. (14) discovered that intrusive and repetitive sexual fantasies were prevalent among individuals with CSBD, which supports classifying CSBD as a specific variant of OCD. Fuss et al. (15) found that CSBD in OCD tends to coexist with other impulsive, compulsive, and mood disorders, supporting its conceptualization as a compulsive–impulsive disorder. Bőthe et al. (16) found that impulsivity, rather than compulsivity, had a stronger correlation with hyper-sexuality in a large community sample. Levi et al. (17) revealed a connection between obsessive-compulsive symptoms and CSBD among individuals using websites to find sexual partners. They also found that impulsivity was linked to increased online pornography consumption, which in turn played a role in the development of CSBD among those who used the internet for pornography. The resulting picture remains ambiguous as to a specific concept that summarizes what is covered under the name CSBD. In the following set of studies, we will examine whether CSB fits the addiction model, namely whether CSB meets the core elements of ‘behavioural addiction’ as described below.

## Addiction and the ICD definition for CSBD

Traditionally, the concept of addiction was used to involve using exogenous substances, namely drugs (e.g. 18, 19). The release of the DSM-5 broadens the definition of addiction by reclassifying and relabelling Pathological Gambling. Previously categorized under impulse control disorders, Pathological Gambling is now recognized as Gambling Disorder (GD) and classified alongside drug addictions under the category of “Substance-Related and Addictive Disorders” (9, 20). A similar transition occurred in the ICD-11 (1), where Pathological Gambling was reclassified under “Disorders due to Addictive Behaviours,” thus acknowledging the presence of behavioural addictions. However, the ICD-11 (1) classified CSBD under impulse control disorders, and the phenomenology described seems to combine those of addiction, impulse control disorders, and compulsive characteristics. The unification of substance addiction and behavioural addiction under the same category has recently been reinforced by findings from Di Carlo et al. (21), who identified a significant association between frequent substance use and high-risk gaming. The authors conclude that there is a common neurobiological vulnerability for both gaming and substance use.

Griffiths (22) has suggested using six components that constitute every addiction, whether related to substances or behaviours. Those components are salience, mood modification, tolerance, withdrawal, conflict, and relapse. Griffiths’ (2005) components overlap with the DSM-5 (9) criteria for GD. Since the only precedent for behavioural addiction is GD, we will use its criteria as a touchstone to evaluate CSBD. Amongst Griffiths’ six components of addiction, the *ICD-11* requires for the diagnosis of CSBD, only ‘salience’ (“repetitive sexual activities becoming a central focus of the person’s life”), conflict (“Neglecting health and personal care or other interests, activities, responsibilities, and continued repetitive sexual behaviour despite adverse consequences”), and relapse (“Numerous unsuccessful efforts to significantly reduce repetitive sexual behaviour”), while ‘mood-modification’, ‘tolerance’ and ‘withdrawal’ are absent. Anecdotal reports based on clinical experience with CSBD patients support the idea of seeing CSBD as an addiction, but as described above the field suffers from a lack of systematic empirical evidence supporting the endorsement of those six components by CSBD patients. Below, we will review the three elements omitted by the ICD i.e., mood modification, tolerance, and withdrawal which are at the core of addiction disorder. We will discuss their meaning, and the current data attributing them to CSBD.

## Mood modification

Mood often plays a pivotal role in substance abuse, with individuals using drugs or alcohol to self-medicate and alleviate emotional distress. The relationship between mood, mental state, and addiction is multifaceted. Individuals with higher levels of alexithymia (difficulty identifying and expressing emotions) and lower interoceptive sensibility (the ability to perceive internal body signals) are more prone to problematic internet use (PUI). This suggests that challenges in emotional awareness and bodily perception may contribute to internet addiction, as those with emotion identification difficulties may turn to the internet for coping (23). Additionally, higher levels of obsessional impulses and impulsivity are significantly associated with increased PUI (24). Mood modifications are not just precursors to substance use or dysfunctional behaviours but are fundamental components of the addiction framework, emerging and evolving as the addiction develops. According to Koob and Volkow, (25), the addiction cycle comprises three stages: binge or intoxication, withdrawal and negative affect, and preoccupation or anticipation. During the withdrawal stage, individuals experience negative emotions like anxiety and dysphoria, which can trigger relapse as they seek relief through substance use.

Studies have shown that adverse emotional states can heighten cravings for substances, such as nicotine, cocaine, alcohol, and heroin (26–29). Behavioural addictions like gambling or hypersexual behaviour may serve as coping strategies for managing unpleasant emotions, as highlighted by Carnes (30) and Griffiths (22). It is argued that individuals with CSBD frequently employ sex as a strategy for mood regulation or coping with negative emotions (31–33).

## Tolerance and incentive-salience

The DSM-5 defines tolerance, as either (a) a need for markedly increased amounts of a substance to achieve intoxication or a desired effect or (b) markedly diminished effect with continued use of the same amount of a substance. Karila et al. (34) describe a few features of CSBD that could suggest tolerance. Coleman-Kennedy and Pendley (35) claim that just as in substance addiction, individuals with CSBD experience the continual need to expand the time spent in sexual activity to ease the emotional pain. The related concept of ‘incentive salience’ suggests that people with addictive disorder experience intensified cravings or desire for addictive stimuli rather than increased pleasure from them, as outlined by Robinson and Berridge (36). This theory suggests that people with addictive disorder are triggered by minor cues associated with addictive stimuli, making the incentive more salient and placing them in a position of more ‘wanting’. Nevertheless, their level of ‘liking’ remains indifferent with not enough enjoyment to satisfy the increased ‘wanting’.

In their research, Brand et al. (37) found that ventral striatum activity, observed during engagement with preferred pornographic images, is correlated with symptoms of Internet pornography addiction. This brain region, recognized for its involvement in reward anticipation and craving, plays a pivotal role in linking the processes of wanting and liking, with incentives calibrated based on their interactive consequences (38–40). Extended drug use, as highlighted by Robinson and Berridge (39), may result in compulsive drug seeking (i.e., wanting) and drug taking without a corresponding enjoyment (i.e., liking) upon consumption.

“It is further proposed that sensitization of the neural systems responsible for incentive salience (for ‘wanting’) can occur independently of changes in neural systems that mediate the subjective pleasurable effects of drugs (drug ‘liking’) and of neural systems that mediate withdrawal. Thus, sensitization of incentive salience can produce addictive behaviour (compulsive drug seeking and drug taking) even if the expectation of drug pleasure or the aversive properties of withdrawal are diminished and even in the face of strong disincentives, including the loss of reputation, job, home and family (39)”. In accordance with this theory, regarding CSBD, Voon et al. (41) reported a dissociation in individuals with problematic pornography use, where a high level of wanting was not necessarily associated with an equivalent degree of liking. In their study, Gola et al. (42) observed a similar pattern, reinforcing the notion of a disconnection between wanting and liking in individuals with problematic pornography use. While some (43) view ‘tolerance’ and ‘incentive salience theory’ as two competing explanations for the need for increasing consumption by people with addictive disorder, Robinson and Berridge (36) see these two mechanisms as complementary and mutually compatible. The literature currently lacks a comprehensive analysis of the relationship between ‘liking’ and ‘wanting’ and their connection to sexual cue-reactivity. While previous studies have primarily concentrated on group differences observed in neuroimaging indices, our study aims to predominantly examine the behavioural response to sexual stimuli. Addressing these gaps through research may provide crucial insights for accurately identifying CSB.

## Withdrawal

Withdrawal symptoms encompass unpleasant physical or emotional sensations that arise upon the discontinuation or reduction of a specific activity or substance (44). In substance use disorder this is characterized by neuro-adaptive changes at the transmitter-receptor level. Withdrawal symptoms in behavioural addictions like pornography watching necessitate a distinct explanation, as they differ from the neuro-adaptive changes seen in substance dependence. Piper (45) suggested that addictive behaviours, such as tobacco (or pornography consumption), become ingrained daily rituals triggered by internal or external circumstances. Avoiding those rituals leading to emotional and somatic withdrawal symptoms particularly when used as a self-regulation method during stressful events. Wray and Dickerson (46) revealed significant mood and behavioural disturbances akin to alcohol addiction during the recovery phase from GD. Subsequent research, including studies by Blaszczynski et al. (47) Cunningham‐Williams et al. (48), and Rosenthal and Lesieur (49), has consistently identified withdrawal phenomena in individuals with GD.

In the context of CSBD, there is a widespread assertion that withdrawal symptoms manifest when individuals attempt to avoid or decrease their sexual activity (e.g., 34, 50–52). Those assertions are often based on general impressions rather than methodological research, suggesting a need for further empirical investigation in this area.

## Rationale and hypotheses

The literature so far describes a debate over whether to classify CSB as an impulse control disorder or as a behavioural addiction. Sassover and Weinstein (13) have presented a debate paper and argued that, due to a lack of validated experiments and data, there is currently insufficient evidence to support CSB as a behavioural addiction. The following studies will examine three components that are essential for a behavioural addiction model, namely mood modification, withdrawal and sensitization or tolerance, in the hope of clarifying the issue of the classification of CSB.

## Study 1

### Rationale and hypotheses

In this study we will induce different mood states and examine the changes it causes to the level of sexual craving and whether it differs between individuals with and without CSB. While positive mood presumed to induce craving for both groups, we postulated that if CSB is a behavioural addiction, mood would affect CSB and non-CSB participants in a different way. We predict higher levels of craving for pornography as a reaction to positive and negative mood induction and that this effect will be only for individuals with CSB and not for those without.

### Method

#### Participants

The Institutional Review Board of Ariel University approved the study, approval number 2020930. The research sample was recruited through open “sexaholic-anonymous” meetings and social media groups. For their participation and time loss, a small monetary compensation was promised. All participants signed an informed consent prior to their participation. Overall, 44 people participated in the study. However, since only one participant identified herself as a woman, she was excluded from the study. Thus, the final sample included 43 participants between the ages of 18-41 with an average age of 25.81 (SD = 5.48), all of whom males, as mentioned. Regarding the participants’ sexual orientation, 30 (69.8%) participants identified as heterosexuals, 7 (16.3%) identified as homosexuals, and 6 (14%) chose not to disclose their orientation. Participants were divided into two groups based on their scores on the Hebrew Bergen-Yale Sex Addiction Scale (HBYSAS). The HBYSAS, which reflects Griffiths (22) six addiction components, assessed sexual behaviours and cognitions over the past year using a five-point Likert scale (0 = very rarely to 4 = very often). Those scoring 18 and above classified as CSB and those scoring below 18 classified as non-CSB. The CSB group consisted of 22 participants with a mean score of (M = 22.64) and a standard deviation of (SD = 2.15). The non-CSB group consisted of 21 participants with a mean score of (M = 9.76) and a standard deviation of (SD = 5.59). Among the participants in the non-CSB group, 3 received a score of 0 and were classified by the questionnaire as “no sex addiction.” 2 participants received a score between 1 and 6 and were classified as “low sex addiction risk,” while 16 participants received a score between 7 and 17 and were classified by the questionnaire authors as “moderate sex addiction risk”. No significant differences between groups were found regarding years of education, F (1, 41) = 0.96, p = .33, η2p = .02 (M = 11.81, SD = 3.31 for non-CSB participants; M = 12.59, SD = 1.71 for CSB participants). The participants allocated to the CSB groups were significantly older (M = 27.59, SD = 4.35) than the non-CSB participants (M = 23.85, SD = 6.01), F (1, 40) = 5.41, p = .03, η2p = .12. In the non-CSB group, 15 participants (71.4% of the group’s participants) identified as heterosexuals, 3 (14.3%) identified as homosexuals, and the rest 3 (14.3%) chose not to identify their orientation. In the CSB group, 15 participants (68.2%) identified as heterosexual, 4 (18.18%) identified as homosexual, and the remaining 3 (13.64%) chose not to identify. The results of the Fisher’s exact test, p = 1, indicated a non-significant association between group affiliation and sexual orientation.

#### Measures

Hebrew Bergen-Yale Sex Addiction Scale (HBYSAS; 53). The HBYSAS questionnaire is the Hebrew version of the Bergen Yale Sex Addiction Scale (BYSAS) developed by Andreassen et al. (54). The BYSAS was based on the six components of addiction proposed by Griffiths (22) as mentioned above. Both the HBYSAS and ICD-11 criteria for CSB focus on uncontrolled sexual behaviour, repeated failed attempts to reduce it, and the negative consequences of such behaviour. However, the HBYSAS uniquely addresses tolerance (increased urges over time) and withdrawal (distress when unable to engage in sexual behaviour), which are not part of the ICD-11 definition. The ICD-11 emphasizes the behaviour’s centrality in the individual’s life and its persistence despite lack of satisfaction, while the HBYSAS includes the use of sex as a coping mechanism for negative emotions. The scale assessed sexual behaviours and cognitions during the past year using a five-point Likert scale ranging from 0 = very rarely to 4 = very often. A sample item asked how often during the past year the participant spent a lot of time thinking about sex, masturbation, or planned sex.

The total score was acquired by summation and ranged between 0 and 24. A total score of 0 is classified as “no risk of sex addiction”, 1 to 6 as “low risk of sex addiction”, 7 to 17 “moderate risk for sex addiction” and score above 18 as “high risk of sexual addiction”. The score of 18 was designated as the cutoff by the questionnaire authors and was used in the current study. According to the authors’ recommendation, a cut-off score of 18 or above was set to ensure relatively high scores (“often” or “very often”) on most items, combined with sufficiently significant scores or at least some presence on the remaining items. Thus, a composite score of 18 or above indicates a more pervasive presence of the symptoms, aligning with the operational definition of addiction in the literature. The original BYSAS presented with good internal consistency (Cronbach’s α =.83), the internal consistency of the HBYSAS was acceptable (Cronbach’s α = 0.79; 53), and in the current study, the internal consistency was excellent (Cronbach’s α = .95).

Positive and Negative Affect Schedule (PANAS; 55). The PANAS is a widely used self-report measure developed to assess two major domains of affect: Positive Affect (PA) and Negative Affect (NA). The PANAS comprises 10 items measuring PA (e.g. alert, inspired, and enthusiastic) and 10 items measuring NA (e.g. distressed, upset, and guilty). Participants rate the degree to which they endorse each item on a five-point Likert scale (1 = very slightly or not at all; 5 = extremely). Items relevant for each affect are summed separately and yield PA and NA total scores. Higher scores represent greater endorsement of the relevant construct.

The validity and reliability of PANAS has been demonstrated in several studies Cronbach’s alpha in the original version was 0.89 for the PA scale and 0.85 for the negative scale (55). In the current study we used the Hebrew version that was developed and validated by Anaby et al. (56). Internal consistency in our study ranged between 0.81 and 0.85 for PA and between 0.76 and 0.80 for the NA.

Pornography Craving Questionnaire (PCQ; 57). The 12-item PCQ measures present craving for pornography thorough five elements (i.e., perceived control over use, current desire to use, psychophysiological reactivity, intentions to use, and mood changes). Sample items include “If the situation allowed, I would watch porn right now” and “If I watched porn right now, I would have difficulty stopping.” Respondents are asked to indicate how strongly they agree with each item using seven points Likert scale, from 1- “disagree completely”, to 7- “agree completely”. The general scores of the PCQ were obtain by summation of the scores of each item, ranging from 12 to 84. Higher scores indicate greater current craving for pornography. The internal consistency for the PCQ was excellent (Cronbach’s α = .91) in the original as well as in subsequent samples (57). The PCQ was also shown to have convergent, criterion, and discriminant validity (58). We translated the questionnaire to Hebrew for the purpose of the current study using a translation back translation method. In the current study, the internal consistency was excellent for all the four times the PCQ was used (Cronbach’s α ranging between.91 and.94).

#### Procedure

All participants filled in their demographics regarding their, age, gender, sexual orientation, and years of education. Then they were asked to fill out the HYBSAS, PCQ and the PANAS for baseline measurement. Participants with a score of 18 or above in the HYBSAS were assigned to the CSB group, and the rest were assigned to the non-CSB group. The PCQ was used to assess the level of craving for pornography, while the PANAS was employed to determine whether the mood induction was successful. The participants underwent mood induction manipulation, by watching three short movies of two minutes length each. One movie included a comic routine to induce pleasant mood; another movie was an interview with relatives to casualties after a terror attack to induce an unpleasant mood; and another movie was a media report regarding a nature resort, in order to induce a more natural mood. The stimulus movies’ order was counterbalanced. After each movie, participants filled out the PCQ and PANAS. After completion, the participants were thanked, debriefed, and were given the promised compensation.

#### Statistical analyses

Prior probing the research question, the manipulation effectiveness was evaluated, by conducting two mixed design 4 by 2 [condition by CSB affiliation] ANOVAs, which in both the condition was the within-subject factor. Each ANOVA was conducted separately for the negative and the positive mood scores obtained by the PANAS. For the research question, a mixed design 4 by 2 [condition by research group] ANOVA was conducted, in which the condition was the within-subject factor, and the average score in the PCQ was the dependent variable. The data were analysed using IBM SPSS Statistics (Version 29). *Post hoc* analyses utilizing paired t-tests with Bonferroni correction were performed for every main or interaction effect. Additionally, for each effect, *post-hoc* power analyses were computed using G*Power software, version 3.1 (59).

### Results

#### Preliminary analyses

Prior to testing the research hypotheses, the manipulation effectiveness was evaluated. This was achieved by conducting two mixed design 4 X 2 [condition X CSB affiliation] ANOVAs, which in both the condition was the within-subject factor. Each ANOVA was conducted separately for the negative and the positive mood scores obtained by the PANAS.

Regarding the positive mood scores, Mauchly’s test indicated that the assumption of sphericity had been retained, *Mauchly’s W* = .78, χ^2^ (5) = 10.11, *p* = .07. The main effect for condition was significant, *F* (3, 123) = 19.48, *p* <.001, η^2^ *_p_* = .32, with power value of 1. *Post hoc* analyses using *t* tests for paired samples with Bonferroni correction (α = .05/6 = .008) showed that the average positive affect score reported after watching the unpleasant movie (*M* = 25.98, *SD* = 8.05) was significantly lower than the other conditions (*M* = 31.44, SD = 5.85 for baseline measurement; *M* = 32.30, *SD* = 6.85 after pleasant movie; *M* = 31.16, *SD* = 6.93 after neutral movie), *t*(42) = -5.01, *p* <.001, Cohen’s *d* = -.76 for the comparison between the average PA score measured after the unpleasant stimulus and the one measured at the baseline level; *t*(42) = -6.33, *p* <.001, Cohen’s *d* = -.96 for the comparison between the average PA score measured after the unpleasant stimulus and the one measured after the pleasant stimulus; and *t*(42) = -6.04, *p* <.001, Cohen’s *d* = -.92 for the comparison between the average PA score measured after the unpleasant stimulus and the one measured after the neutral stimulus. The comparison between the average PA score measured at the baseline and the one measured after the positive stimulus was non-significant, *t*(42) = -1.12, *p* = .27, Cohen’s *d* = -.17, as well the comparison between the average PA score measured at the baseline and the one measured after the neutral stimulus, *t*(42) = 0.30, *p* = .76, Cohen’s *d* = .05. The comparison between average PA score measured after the neutral stimulus and the score measured after the pleasant stimulus proved also to be non-significant, *t*(42) = -1.47, *p* = .15, Cohen’s *d* = -.22.

**Figure 1:**
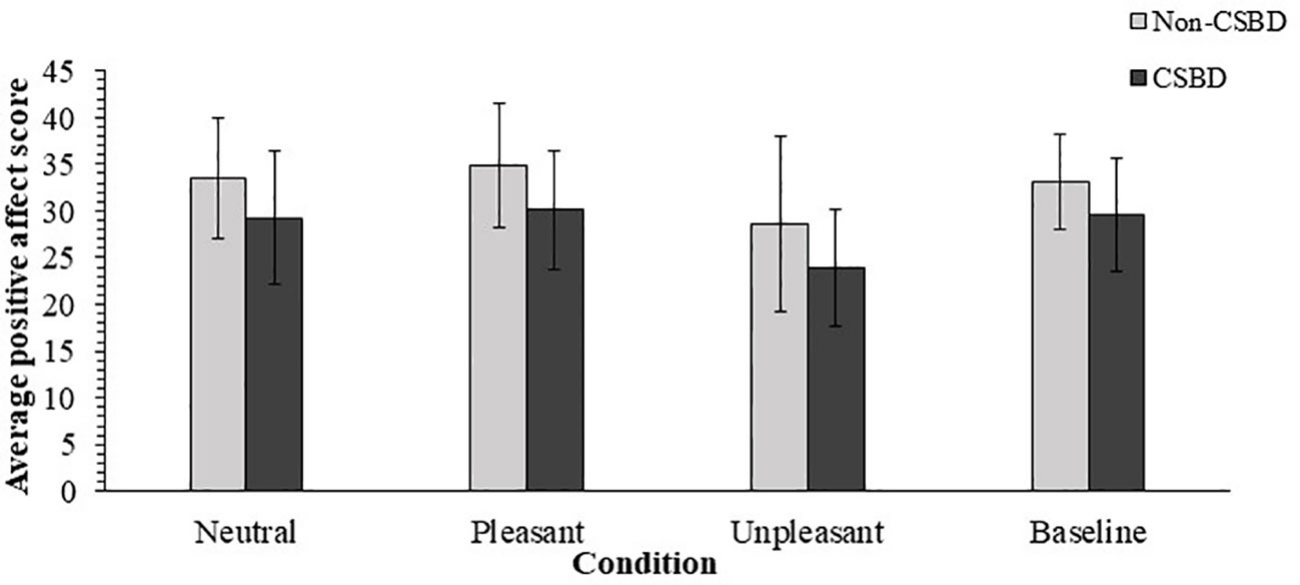
Average PANAS Positive Affect Scores by Mood Induction Conditions. CSBD, compulsive sexual behavior disorder (error bars show standard deviations).

The analysis also showed main effect for the between subjects factor, *F* (1, 41) = 5.51, *p* < 0.05, η^2^ *_p_* = .12, with power value of.90, meaning that non-CSB participants reported for a significantly more positive affect in average (*M* = 32.29, *SD* = 6.08), compared to CSB participants (*M* = 28.25, *SD* = 5.18). No significant interaction of condition X group was found, *F* (3, 123) = 0.08, *p* = .97, η^2^ *_p_* = .002, with power value of.04. The results are illustrated in **Figure 1**.

Regarding the negative mood scores, Mauchly’s test of sphericity was non-significant, *Mauchly’s W* = .80, χ^2^ (5) = 8.72, *p* = .12. The mixed design ANOVA yielded a significant main effect for condition, *F* (3, 123) = 3.00, *p* <.05, η^2^ *_p_* = .07, with power value of 1. *Post hoc* analyses using *t* tests for paired samples with Bonferroni correction (α = .05/6 = .008) showed a trend toward significance when the average NA score measured during baseline measurement (*M* = 22.91, *SD* = 7.79) and the one measured after the neutral stimulus (*M* = 19.98, *SD* = 4.82), with the baseline score was higher, *t* (42) = 2.60, *p* = .01, Cohen’s *d* =.40.

The rest of the comparisons were non-significant. Specifically, The comparison between the average NA score measured after the unpleasant stimulus (*M* = 22.37, *SD* = 7.64) was not significantly different than the one measured at baseline, *t*(42) = -0.40, *p* = .69, Cohen’s *d* = -.06; than the one measured after the positive stimulus (*M* = 20.53, *SD* = 7.85), *t*(42) = 1.53, *p* = .13, Cohen’s *d* = .23; or from the one measured after the neutral stimulus (*M* = 19.98, *SD* = 4.82), *t*(42) = 2.36, *p* <.05, Cohen’s *d* = .36. Non-significant resulted also emerged when the average NA score, measured after the positive score was compared with either the score measured at baseline, *t* (42) = 2.39, *p* <.05, Cohen’s *d* = .37; or the score measured after the neutral stimulus, *t* (42) = -0.49, *p* = .63, Cohen’s *d* = -.08.

The ANOVA revealed a significant main effect for the between-subjects factor, *F* (1, 41) = 15.46, *p* <.001, η^2^ *_p_* = .27, with power value of 1, meaning that the participants who affiliated with CSB reported an average score of NA (*M* = 24.22, *SD* = 4.82), which was higher from the scores of the participants who had not CSB (*M* = 18.55, *SD* = 4.62). No significant interaction of condition X CSB affiliation was found, *F* (3, 123) = 0.56, *p* = .65, η^2^ *_p_* = 0.01 with power value of.12.

#### Analysis

A mixed design 4 X 2 [condition X research group] ANOVA was performed, in which the condition was the within-subject factor, and the average score in the PCQ was the dependent variable. Mauchly’s test of sphericity was non-significant, *Mauchly’s W* = .86, χ^2^ (5) = 6.16, *p* = .29. A significant main effect for the research groups was found, *F* (1, 42) = 65.68, *p* <.001, η^2^ *_p_* = .62, with a power value of 1, meaning that the average PCQ score for the participants in the CSB group was higher than the score of the participants in the non-CSB group. The means and standard deviations regarding this main effect are listed in **Table 1**, presented in the next page.

**Table 1:** Means and standard deviations of PCQ scores for research groups by experimental condition.

Stimulus Type	CSBD (*n* = 22)		Non-CSBD (*n* = 21)		Total (*N* = 43)	
	*M*	*SD*	*M*	*SD*	*M*	*SD*
Baseline	52.32	11.67	25.90	11.94	39.42	18.45
Pleasant	47.91	8.61	21.19	8.82	34.86	17.42
Unpleasant	38.45	8.81	19.24	8.99	29.07	14.08
Neutral	49.50	9.91	22.57	10.14	36.35	17.56
Total	47.05	10.68	22.23	9.32	34.92	16.00

*M*, Mean; *SD*, Standard deviation; PCQ, Pornography craving questionnaire; CSBD, compulsive sexual behavior disorder.

The ANOVA yielded also a significant main effect for condition, *F* (3, 123) = 20.09, *p* <.001, η^2^ *_p_* = .33 with a power value of 1. *Post-hoc* analyses of the simple effects, using *t* tests for paired samples with Bonferroni correction (α = .05/6 = .008) revealed that the average PCQ score obtained during the baseline measurement was significantly higher than the average score obtained after both the pleasant stimulus, *t* (42) = 3.27, *p* <.01, *Cohen’s d* = .50; and the unpleasant stimulus, *t* (42) = 6.34, *p* <.001, *Cohen’s d* = .97. However, the comparison between the average PCQ score obtained during the baseline measurement and the average score measured after the neutral stimulus was not significant, *t*(42) = 2.17, *p* <.05, *Cohen’s d* = .33. *Post-hoc* analyses revealed also that the PCQ average score, measured after the presentation of a pleasant stimulus was significantly higher than the score obtained after the unpleasant stimulus, *t*(42) = 4.28, *p* <.001, *Cohen’s d* = .65, but not significantly different from average score obtained after the neutral stimulus, *t*(42) = -1.13, *p* = .26, *Cohen’s d* = -.17. Finally, the *post-hoc* analyses revealed that the PCQ average score, measured after the neutral stimulus were significantly higher than the score obtained after the unpleasant stimulus, *t*(42) = -5.73, *p* <.001, *Cohen’s d* = -.87. The means and standard deviations regarding this main effect are also listed in **Table 1**.

Lastly, the ANOVA revealed a significant condition by CSB interaction effect, *F* (3, 123) = 3.80, *p* = 0.01, η^2^ *_p_* = .09 with a power value of.76. *Post-hoc* analyses of the simple effects, using *t* tests for paired samples with Bonferroni correction (α = .05/12 = 0.004), revealed that regarding the participants who did not qualify for CSB, only one comparison was significant: The average PCQ score measured during baseline was higher than the score measured after the unpleasant stimulus, *t*(20) = 4.09, *p* <.001, *Cohen’s d* = .89. The average PCQ score measured during baseline was not significantly different than PCQ score measured either after the pleasant or the neutral stimulus, *t* (20) = 3.05, *p* <.01, *Cohen’s d* = .67; and *t* (20) = 1.90, *p* = .07, *Cohen’s d* = .42 accordingly. A non-significant difference was found between the average PCQ scores measured after the pleasant stimulus and the score measured after both the unpleasant stimulus, *t*(20) = 2.85, *p* = .01, *Cohen’s d* = .62; and the neutral stimulus, *t*(20) = -1.39, *p* = .18, *Cohen’s d* = -.30. Finally, for the non-CSB participants, A non-significant difference between the average PCQ scores measured after the unpleasant stimulus and the scores measured after the neutral stimulus, *t*(20) = -3.08, *p* = .01, *Cohen’s d* = -.67.

**Figure 2:**
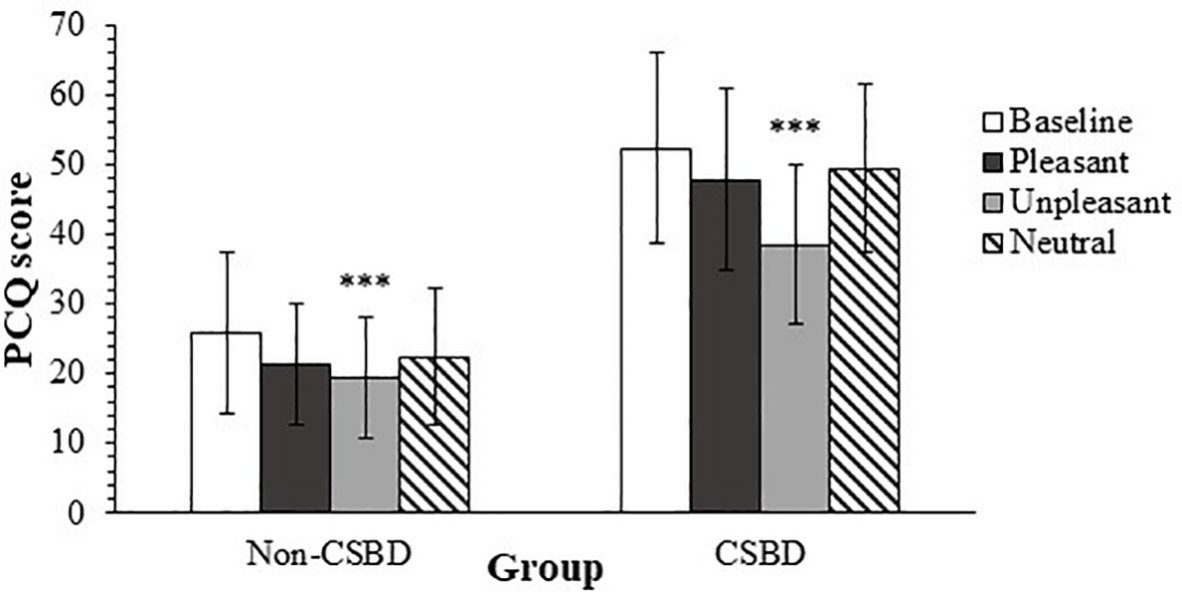
Average PCQ Scores by CSBD diagnosis for Each Mood Induction Condition. PCQ, pornography craving questionnaire; CSBD, compulsive sexual behavior disorder (error bars show standard deviations). ***p <.001.

For the participants who qualified to CSB, *post-hoc* analyses revealed that the average PCQ score measured the unpleasant stimulus was significantly lower than the average score obtained after each of the three other conditions – pleasant stimulus, *t*(21) = 4.06, *p* = .001, *Cohen’s d* = .87; neutral stimulus, *t*(21) = -5.63, *p* <.001, *Cohen’s d* = -1.20; and baseline measurement, *t*(21) = 5.32, *p* <.001, *Cohen’s d* = 1.13. The remaining *post-hoc* comparisons of the average PCQ score did not demonstrate a significant difference. Specifically, the difference between the average PCQ score obtained during baseline was not statistically different than the scores obtained both after the pleasant and the neutral stimulus, with values of *t*(21) = 1.89, *p* = .07, *Cohen’s d* = .40; and *t*(21) = 1.26, *p* = .22, *Cohen’s d* = .27 accordingly to each comparison. The comparison between the average PCQ scores obtained after the pleasant and neutral conditions proved to be non-significant as well, *t* (21) = -0.66, *p* = .52, *Cohen’s d* = -.14. The means and standard deviations regarding the interaction’s simple effects are also listed in **Table 1**, mentioned above. The results are illustrated in **Figure 2**.

### Discussion

The findings of this study indicate a discernible causal link between positive affect and pornography craving (PC) in both groups. Furthermore, both CSB and non-CSB individuals exhibited reduced PC following exposure to the negative movie. This reduction was statistically significant for the non-CSB group when compared to baseline. However, for the CSB group, this effect was significant not only compared to baseline but also in comparison to other conditions. Moreover, the impact of the negative movie was notably more pronounced in the CSB group, leading to PC levels that were no longer higher than those of the non-CSB group.

The positive and negative affect scales (PA, NA) as represented in the PANAS are mutually independent and can represent two different aspects of bad mood (55). Low PA is traditionally associated with a lack of liveliness, loss of pleasure, and depressed experience, while NA represents irritability and nervousness as reflected in an anxious state of mind (55, 60). The current research does not allow us to draw conclusions regarding the relationship between an anxious mood and PC, However, it can be demonstrated that a low PA leads to a decrease in PC in both groups.

Inducing depression has already been found to reduce sexual desire (61). Previous studies using PANAS in general population have all shown that low PA is associated with lowered sexual desire. The findings regarding NA are less conclusive but all indicate a weak to no relationship or even a positive relationship so that anxiety leads to sexual interest (62–65). Grov et al. (66) examined the relationship between mood and sexual interest using self-report diaries, and additionally compared between CSB and non-CSB individuals. Grov et al. (66) found that anxiety and nervousness were associated with increase in sexual activity, particularly among those without CSB. Like in our study, Grov et al. (66) found that depression and discouragement were associated with reduced sexual activity for both groups but unlike our study the effect was less pronounced for the CSB group. Grov et al. (66) assumed that the depressive effect is less relevant to CSB individuals due to their pervasive sexual desire. It is important to note that Grov’s (2010) study dealt with sexual activity with other partners while our study dealt with watching pornography. Sexual activity can entail a wide range of emotions. Out of anxiety and nervousness, sex can have a calming effect of sexual release as well as possible “excitation transfer” of the anxious arousal. In depressed mood, seeking sexual activity can be utilized for intimacy and self-assurance (67). It might be intuitively argued that pornography provides no intimacy and therefore in our study low positive mood diminished PC to the minimum level for both groups, CSB and non-CSB participants. The same idea can be found by Grov et al. (66) that searching multiple partners was associated with elevated mood among non-CSB individuals and associated with depressive mood among CSB individuals.

To sum up, our study, like previous studies (68) shows that the relationship between mood and sexual activity takes different shapes depending on the specific mood, the specific sexual act. At the same time, the extent to which this relationship between mood and sexual activity distinguishes between CSB and non-CSB remains unproven.

Lastly, the results of the experiment indicate that the highest level of craving for pornography occurred in the initial state, where participants had not watched any films. It is possible that the absence of external stimuli in the initial state might have led participants to focus more on their internal desires and thoughts, thus intensifying their cravings. When occupied with watching films, their attention would have been partially diverted, potentially diminishing the immediate intensity of their cravings for pornography.

A significant limitation lies in the fact that, while the experiment specifically examined the impact of mood on the desire for pornography, the grouping of participants and the data collected do not provide information specific to pornography-related sexual behaviour but rather assess sexual behaviour in general.

## Study 2

### Rationale and hypotheses

Due to the lack of empirical studies examining the presence of tolerance and incentive salience, our aim in this study is to investigate ‘wanting’ and ‘liking’ in CSB and non-CSB participants. If CSB is a behavioural addiction, individuals with CSB should report more subjective ‘wanting’ and less ‘liking’ in response to increased explicit sexual stimuli compared with individuals without CSB.

### Method

#### Participants

The Institutional Review Board of Ariel University approved the study, approval number 2020930. The sample was recruited through social media groups. All participants signed an informed consent prior to their participation. The sample included 92 participants between the ages of 18-65, with an average age of 30.76 (*SD* = 11.52). 80 participants (87%) identified as males, 4 (4.3%) as females, and the rest 8 (8.7%) identified with other genders. Regarding the participants’ sexual orientation, 64 (69.6%) participants identified as heterosexuals, 4 (4.3%) identified as homosexuals, and 24 (26.1%) chose not to disclose their orientation. The majority (78%) of the sample had at least 12 years of education.

Participants were divided into two groups based on their scores: those scoring 18 and above (classified as CSB) and those scoring below 18 (classified as non-CSB). The CSB group consisted of 44 participants with a mean score of (M =20.61) and a standard deviation of (SD =1.97). The non-CSB group consisted of 48 participants with a mean score of (M =10.60) and a standard deviation of (SD =5.65). Among the participants in the Non-CSB group, 4 received a score of 0 and were classified by the questionnaire as “no sex addiction.” 7 participants received a score between 1 and 6 and were classified as “low sex addiction risk,” while 37 participants received a score between 7 and 17 and were classified by the questionnaire authors as “moderate sex addiction risk”. No significant between-group differences were found regarding years of education, *F*(1,90) = 0.24, *p* = .62, η^2^ *_p_* = 0 (*M* = 12.63, *SD* = 2.99 for non-CSB participants; *M* = 12.34, *SD* = 2.48 for CSB participants), or regarding their age, *F*(1,90) = 1.12, *p* = .29, η^2^ *_p_* = .01 (*M* = 31.98, *SD* = 12.09 for non-CSB participants; *M* = 29.43, *SD* = 10.85 for CSB participants). Within the non-CSB group, 41 participants (85.4%) identified as males, 3 (6.3%) as women, and the rest 4 (8.3%) chose not to disclose their gender. 39 participants (88.6%) who were assigned to the CSB group identified as men, 1 participant (2.3%) as a woman, and the rest 4 (9.1%) chose not to identify their gender. The results of Fisher’s exact test, *p* = .80, indicated a non-significant association between group assignment and gender. A non-significant association was also found in the association between group allocation and sexual orientation, as indicated by Fisher’s exact test, = .09. Specifically, within the non-CSB group, 34 participants (70.8%) identified as heterosexuals, 4 (8.3%) – as homosexuals, with the rest 10 (20.8%) not identifying themselves. Within the CSB group, 30 (68.2%) participants identified as heterosexuals, and the rest (14 participants, 31.8%) chose not to identify themselves.

#### Watching materials

Initially, three different masturbation videos were selected. Each video was divided into three one-minute clips, varying in the level of explicit content. The first clip showed a woman fully clothed, engaging in self-rubbing motions with sexual connotations. In the second clip, the same woman was dressed in lingerie (bra and panties), continuing to rub herself and directing her actions toward more private areas. The third clip depicted the woman completely nude, masturbating. Each participant viewed all three clips from the same video in sequential order from the least explicit to the most explicit. The assignment of which video each participant received was randomized.

#### Measures

HBYSAS (53). Details about the HBYSAS were mentioned in Study 1. In the current study, the internal consistency was good (Cronbach’s α = .83).

#### Procedure

Participants filled in demographic details on a designated questionnaire, which was the same as the first study. Afterwards, they completed the HBYSAS, and as in the first study, a total score of 18 or above was set as the criteria for assignment under the CSB group. Then, participants were explained that they would be presented with an erotic movie divided into three segments with an increasing level of explicitness. Before viewing the first segment, the participant reported their desired level for consuming videotaped pornography in the present moment on a scale from 1 to 100. After watching each video segment, the participants rated both their level of enjoyment (liking measurement) and their desire to watch erotic movies (wanting measurement) on a scale ranging from 1 to 100, hence rating the liking measurement three times and the wanting measurement four times.

#### Statistical analyses

For the research question, a mixed design 3 by 2 [Liking measurement by research group] ANOVA and a mixed design 4 by 2 [Wanting measurement by research group] ANOVA were conducted, both in which the condition was the within-subject factor and the wanting or liking scores served as the dependent variables. The data were analysed using IBM SPSS Statistics (Version 29). Additionally, for each effect, *post-hoc* power analyses were computed using G*Power software, version 3.1 (59).

### Results

The second research question postulated whether the attraction towards pornography, measured by either liking or wanting it, would differ by CSB status. Regarding the liking measurement, a 3 by 2 [level of movie explicitness by CSB affiliation] mixed-design ANOVA was conducted, with the level of explicitness as the within-subject factor. Mauchly’s test of sphericity was non-significant, *Mauchly’s W* = 1, χ^2^ (2) = 0.25, *p* = .88.

**Figure 3:**
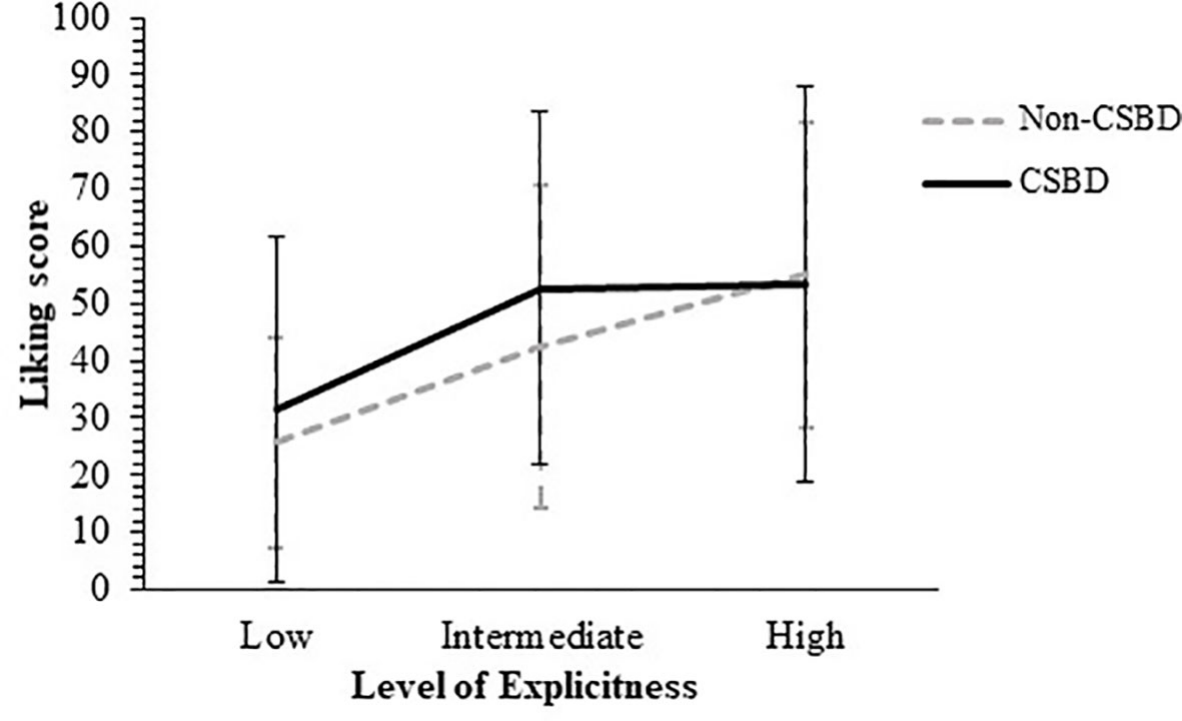
Average Wanting Scores by CSBD diagnosis According to the Movie Explicitness Level. CSBD, compulsive sexual behavior disorder (error bars show standard deviations).

The ANOVA yielded a significant main effect of the level of movie explicitness, *F* (2, 180) = 30.62, *p* <.001, η^2^ *_p_* = .25, with a power value of 1. Non-significant effect for CSB affiliation was found, *F* (1, 90) = 1.14, *p* = .29, η^2^ *_p_* = .01, meaning that the average liking score of the non-CSB participants (*M* = 41.07, *SD* = 19.07) was not significantly different than the average liking score of the CSB participants (*M* = 45.84, *SD* = 23.77). Finally, no significant interaction effect of levels of explicitness by CSB affiliation was found, *F* (2, 180) = 1.52, *p* = .22, η^2^ *_p <_* 0.05, with a power value of.38. *Post hoc* analyses for the explicitness level were conducted using *t* tests for paired samples with Bonferroni correction (α = .05/3). The analyses revealed that the average liking score obtained after watching the movie with the lowest explicitness level (*M* = 28.46, *SD* = 24.78) was lower than the average liking scores obtained after watching both the movie with intermediate explicitness level (*M* = 47.36, *SD* = 29.91), *t*(1, 91) = -5.73, *p* <.001, *Cohen’s d* = -.60; and high explicitness levels (*M* = 54.24, *SD* = 30.67), *t*(1, 91) = -7.49, *p* <.001, *Cohen’s d* = -.78. A non-significant difference was found between the liking scores of the movies with intermediate and higher explicitness levels, *t* (1, 91) = -1.98, *p* = .05, *Cohen’s d* = -.21. **Figure 3** shows average liking scores by CSB affiliation according to movie explicitness level.

Regarding the wanting measurement, a 4 by 2 [level of explicitness by CSB affiliation] mixed-design ANOVA was conducted, with the level of explicitness as the within-subject factor. Mauchly’s test of sphericity was non-significant, *Mauchly’s W* = .91, χ^2^ (5) = 7.96, *p* = .15.

The ANOVA revealed a significant effect for CSB: *F* (1, 90) = 6.42, *p* = .01, η^2^ *_p_* = .07, with a power value of.98, meaning that the average wanting score of the non-CSB participants (*M* = 48.58, *SD* = 23.01) was lower than the average wanting score of the CSB participants (*M* = 59.47, *SD* = 24.30). The ANOVA yielded a significant main effect regarding the level of explicitness: *F* (3, 270) = 22.93, *p* <.001, η^2^ *_p_* = .20, with a power value of 1. No significant interaction effect was found for explicitness by CSB affiliation was found, *F* (3, 270) = 0.96, *p* = .41, η^2^ *_p_* = .01, with a power value of.25.

**Figure 4:**
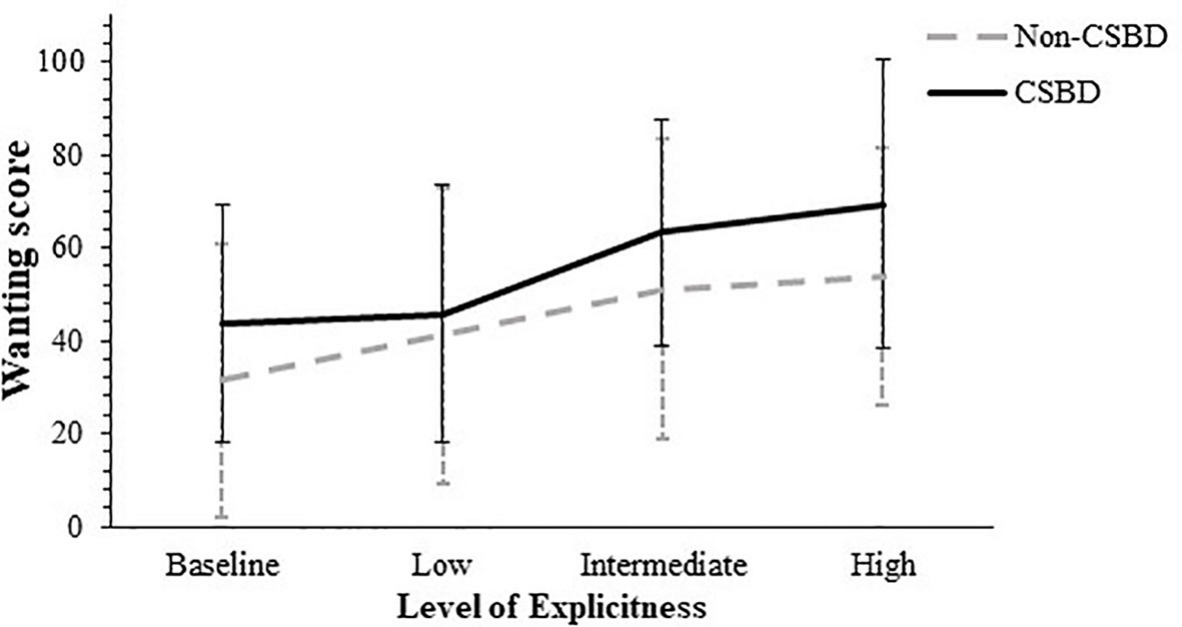
Average Wanting Scores by CSBD diagnosis According to the Movie Explicitness Level. CSBD, compulsive sexual behavior disorder (error bars show standard deviations).

*Post-hoc* analyses for the explicitness level were conducted, using *t* tests for paired samples with Bonferroni correction (α = .05/6). These analyses revealed that the average wanting score obtained prior to watching any movie (*M* = 37.67, *SD* = 28) was lower than the average scores obtained after watching the movies with either intermediate (*M* = 56.91, *SD* = 28.99), *t*(1, 91) = -5.71, *p* <.001, *Cohen’s d* = -.60; or higher explicitness levels (*M* = 61.14, *SD* = 30.49), *t*(1, 91) = -7.17, *p* <.001, *Cohen’s d* = -.75. No significant differences were detected between the average wanting score obtained prior to watching any movie and the average wanting score measured after the movie with the lowest explicitness level (*M* = 43.32, *SD* = 29.52), *t*(1, 91) = -1.58, *p* = .12, *Cohen’s d* = -.16. The average wanting score measured after the movie with the lowest explicitness level was lower than the average scores obtained after watching with either intermediate explicitness level, *t*(1, 91) = -4.52, *p* <.001, *Cohen’s d* = -.47; or higher explicitness levels, *t*(1, 91) = -5.29, *p* <.001, *Cohen’s d* = -.55. No significant differences were found between the scores obtained after watching the movies with intermediate or higher explicitness levels, *t*(1, 91) = -1.43, *p* = .16, *Cohen’s d* = -.15. **Figure 4** shows average wanting scores by CSB affiliation according to movie explicitness level.

### Discussion

Our hypotheses in this study were based on the theory that there are two parallel processes in addiction, i.e., incentive-salience and tolerance. We postulated that if CSB is an addiction, increased explicitness of sexual content would trigger more “wanting” for both groups and that this effect would be significantly higher for the CSB group due to their sensitized system. We also postulated that in response to the same level of sexual stimuli, CSB individuals will feel less satisfaction, namely less ‘liking’, compared to control participants due to tolerance. Regarding the ‘wanting’ hypothesis, our results show that CSB individuals do experience more wanting than non-CSB individuals, but that the difference between groups was not affected by explicitness level, both groups showed elevated wanting in response to more explicit content. As for the ‘liking’ hypothesis, our results show no more ‘liking’ in CSB individuals compared with non-CSB individuals and no increase in liking as a function of increased explicitness in CSB. We found that both groups showed the same pattern of change across levels of explicitness, namely an increase in liking and wanting as the sexual material became more explicit. Our results are supportive of those reported by Voon et al. (41) who identified a similar dissociation between wanting and liking in CSB individuals. They based their analysis on the incentive sensitization theory proposed by Robinson and Berridge (39) who argued for a dissociation between wanting and liking in addiction. According to their theory, as addiction progresses, individuals desire rewards but do not find them pleasurable. However, the self-reported data of their study demonstrated a linear regression between wanting and liking that was not significantly different for CSB and non-CSB. In our study, participants wanted pornography, although their wanting did not increase as a function of the level of explicitness. They did not differ from control participants in their rating of their liking nor was there an interaction between liking and increased level of explicitness in CSB.

There are other studies that have investigated the relationships between wanting and liking in CSB. Gola et al. (42) suggested a potential discordance between liking and wanting in CSB. Their results based heightened expectations for erotic cues among individuals with CSB compared to those without, but they found no between group differences in the rated hedonic value of the actual stimuli presented later. Although sexual cues might elicit greater desire in CSB individuals it does not mean that they would experience higher satisfaction with any form of sexual stimuli. In Gola et al. (42) study, the delay and the limited sexual stimuli might have been disappointing in a way for the CSB group.

A more recent study, File et al. (69) has also studied the mechanism of incentive sensitization focusing on substance use and potentially problematic behaviours. Their study is built upon the notion put forth by Berridge et al. (70) that vivid imagery of reward could evoke measurable properties without the presence of the actual stimuli. For instance, participants were prompted to envision scenarios involving their preferred alcoholic beverage, gambling or sexually active. They employed self-reported measures of ‘expected affective states’ (liking) and levels of ‘self-regulatory effort required to resist or terminate participation in behaviour’ (wanting). The rate of ‘wanting minus liking’ (WML) liking used as an imbalance of the two components and an indicator of incentive salience. The regression models revealed significant relationships between usage frequency and the amount of WML, a marker of incentive sensitization. The findings of File’s study suggest that the sensitization process is more pronounced in substance consumption, although it also manifests in behavioural addictions as well. File et al. (69) results seemingly contradict our findings but it’s crucial to note that our study offer a significant contribution by operationalizing desired behaviours rather than relying solely on imagination. Additionally, our approach differs in that we don’t place all participants on the same continuum. Instead, we identified CSB users compared to non-CSB users, utilizing a cross-sectional study design.

Another issue in the relationship between wanting and liking is that an intense craving for sex or deriving pleasure from sexual experiences, may not cause disturbances in the reward system, and does not necessarily result in a psychopathological condition. The problem may occur, for instance, if there is a decline in the individual’s capacity to derive enjoyment from the activity or if there is an imbalance between liking and wanting (71, 72). In our study, both groups showed a moderately positive correlation between liking and wanting, so that there is no evidence for imbalance between wanting and liking. Finally, more wanting in cases of addiction is not exclusively explained by a sensitized reward system. According to the cue reactivity theory, various natural cues are transformed into conditioned cues that trigger desire and wanting as conditioned responses (73). According to this view, our results might still be explained within the realm of addiction since, in this research, the sexual content was overt without much of a neutral stimulus. Clearly, further research is required to determine whether this process also occurs in CSB.

We used the fixed sequence to measure the level of desire for more explicit content. However, one limitation of the current study is the potential confounding of the condition “explicitness” with the position of the presentation of the different film segments. Each participant viewed the clips in a fixed sequence from the least explicit to the most explicit. This sequential order introduces the possibility that differences between the conditions could be attributed to position effects rather than the explicitness of the content alone. For example, participants may have experienced increasing arousal or habituation effects simply due to the progression of time and continued exposure, regardless of the explicitness of the content. Future studies should consider counterbalancing the order of the clips to control for these potential position effects, allowing for a more accurate assessment of the impact of explicitness on participant responses. Similarly to the first study, another limitation is that although the experiment focused on the desire for pornography as the dependent variable, the participant groupings and the data collected did not isolate pornography-specific sexual behaviour but rather evaluated sexual behaviour more generally.

## Study 3

### Rationale and hypotheses

To the best of our knowledge, there is no prospective research that has examined the effects of abstaining from sexual activity for individuals with CSB. Recently, Lewczuk et al. (74) published a study that investigated the specific impact of abstaining from pornography, but like many other studies, it relies on retrospective self-reports. The purpose of this study is to follow individuals with and without CSB during a 10-day period of abstinence from any sexual activity. In view of previous evidence of habituation of withdrawal symptoms during abstinence from drugs, we expected to see a gradual reduction in withdrawal symptoms in the days of sexual abstinence and to find this effect amongst the CSB group only.

### Method

#### Participants

The Institutional Review Board of Ariel University approved the study, approval number 2020930. The sample was recruited through social media groups. All participants signed an informed consent prior to their participation.

Participants were divided into two groups based on their HBYSAS scores: those scoring 18 and above (classified as CSB group) and those scoring below 18 (classified as non-CSB group). All participants in the study were male. The CSB group consisted of 14 participants with a mean score of (M =20.36) and a standard deviation of (SD =2.24). The non-CSB group consisted of 16 participants with a mean score of (M =10) and a standard deviation of (SD =3.74). Among the participants in the Non-CSB group, none of the participants received a score of 0 and were classified by the questionnaire as “no sex addiction.” 3 participants received a score between 1 and 6 and were classified as “low sex addiction risk,” while 13 participants received a score between 7 and 17 and were classified by the questionnaire authors as “moderate sex addiction risk”. No significant differences between groups were found regarding either years of education, *F*(1,28) = 1.83, *p* = .19, η^2^ *_p_* = .06 (*M* = 12.50, *SD* = 2.22 for non-CSB participants; *M* = 13.71, *SD* = 2.70 for CSB participants), or age, *F*(1,28) = 0.52, *p* = .48, η^2^ *_p <_* 0.05 (*M* = 29.50, *SD* = 9.67 for non-CSB participants; *M* = 31.93, *SD* = 8.62 for CSB participants). Fisher’s exact test showed a significant association between group assignment *p <*0.01. Specifically, within the non-CSB group, all 13 participants (100%) who disclosed their sexual orientation identified themselves as heterosexuals, while in the CSB group, 9 participants (64.3%) identified as heterosexuals, and the rest 5 (35.7%) – as homosexuals.

Out of the 38 participants who initially provided reports, one participant failed to report beyond the baseline, and three others discontinued reporting after the third day. All four of these participants were non-CSB and were excluded from the analyses. At each assessment point, participants were asked to indicate whether they had successfully avoided engaging in sexual activity, with the assurance that their response would not impact their participation reward. Four participants (one with CSB and three without CSB) reported one or two instances of failure and were similarly excluded from the analyses. Therefore, the final analysis comprised 30 participants, 16 without CSB, and 14 with CSB. The age of the sample ranged between the ages of 19-43, with an average age of 30.76 (*SD* = 9.12). Regarding the participants’ sexual orientation, 22 (73.3%) participants identified as heterosexuals, 5 (16.7%) identified as homosexuals, and the rest 3 (10%) chose not to disclose their orientation.

#### Measures

HBYSAS (53). Details about the HBYSAS were mentioned in Study 1. In the current study, the internal consistency was good (*Cronbach’s* α = .88).

The Short Depression, Anxiety, and Stress Scales (DASS-21; 75). The DASS-21 comprises three self-report scales constructed to estimate emotional states associated with depression, anxiety, and stress. Each scale consists of seven items categorized into subscales with analogous content. The depression scale evaluates dysphoria, hopelessness, devaluation of life, self-deprecation, lack of interest/involvement, anhedonia, and inertia. The anxiety scale appraises autonomic arousal, skeletal muscle effects, situational anxiety, and the subjective experience of anxious affect. The stress scale is attuned to chronic, nonspecific arousal levels, encompassing difficulty relaxing, nervous arousal, and tendencies to be easily upset/agitated, irritable/over-reactive, and impatient.

Participants utilized a 4-point Likert scale to rate their agreement with each item and the extent to which the item described their status in the preceding week (0 = not applicable, 3 = highly applicable). Depression, anxiety, and stress scores were derived by summing the ratings for the seven relevant items, yielding scores between 0 and 21. The present study employed a total scale score. The DASS-21 total scale exhibited excellent internal consistency (Cronbach’s α = .93; 76), and its score interpretations demonstrated robust construct validity (76, 77). The Hebrew version, translated by Dr. Janine Lurie, maintained internal consistency levels in both past studies (e.g., 78) and the current research (Cronbach’s α ranged from.75 to.93).

Withdrawal symptoms questionnaire (WSQ). A list of 20 symptoms typically associated with the withdrawal phenomenon was aimed at assessing it specifically regarding sexual addiction. Since there was no designated questionnaire measuring a specific addiction, we searched for existing withdrawal scales developed for other addictions. We found six withdrawal measuring tools that are validated and commonly used. Those are: (1) A questionnaire that measures withdrawal from highly processed food (79); (2) A questionnaire that measures withdrawal from gambling (46); (3,4) Two questionnaires that measure withdrawal from cannabis (80, 81); (5) A questionnaire that measures withdrawal from smoking (82); and (6), a questionnaire that measures withdrawal from excessive social media use (83).

Unifying the items from all questionnaires has revealed six main categories or fields the questionnaires are dealing with: (1) sleep (e.g. My sleep has been troubled); (2) anxiety, tension, and restlessness (e.g. I have been tensed or anxious); (3) physical symptoms (e.g. Felt weak/tired); (4) mood (e.g. I have felt sad or depressed); (5) concentration and cognition (e.g. It has been difficult to think clearly); and (6) craving (e.g. I have had frequent urges to smoke). The total number of items from all six questionnaires was 159. We have identified multiple identical and repetitive items, and we have omitted the replications. We have also excluded the items relating to the craving realm, since craving is not defined as part of withdrawal phenomena, neither in the DSM nor in the ICD. Finally, we chose from each one of the remaining categories 2-3 items, which brought the questionnaire to 20 items.

Participants were asked to indicate on a 5-point Likert scale how a given description reflects their feelings in the last few days, from 1-not at all to 5-in an extreme way. The internal consistency in all four measurement times ranged between good (*Cronbach’s* α = .82) and excellent (*Cronbach’s* α = .93).

#### Procedure

Participants filled in their demographics regarding age, years of education, gender, and sexual orientation, as in the previous studies. Overall, during the experiment, the participants were evaluated at 4 time points – the initial day of their participation, the 3^rd^, the 7th, and the 10th days from their initial date of participation. The 10-day duration was set due to the literature dealing with withdrawal symptoms from either substance or behavioural addictions, revealing that withdrawal symptoms have a time-course of high pick between 2-7 days from cessation (49, 84, 85). In order to avoid biases, participants were assured that they would be given the promised compensation regardless of their realization of remaining sexually abstinent.

On the initial day of participation, participants were guided to abstain from sexual activity for the next 10 days. The participants then completed the HBYSAS, the DASS-21, and the WSQ. As in the above studies, the participants’ HYBSAS score (18 and above versus 17 and lower) determined their allocation to either the CSB or the control groups. The DASS-21 and the WSQ scores at that time point were used for establishing baselines. In the remaining three time points, participants were asked whether they succeeded in remaining abstinent sexually and then completed the DASS-21 and the WSQ.

#### Statistical analyses

Two analyses were conducted to examine differences between individuals who successfully abstained from sexual activity and those who did not in their HBYSAS, WS, and DAAS-21 baseline scores. For the CSB group, Mann-Whitney *U* tests revealed no significant differences between those who abstained and those who did not across all three variables. Specifically, no significant difference was found for HBYSAS, *U* = 7.5, *p* = 1.00; WS, *U* = 11.0, *p* = 0.42; or DAAS-21, *U* = 14.0, *p* = 0.13. Similarly, in the non-CSB group, Mann-Whitney *U* tests indicated no significant differences across the three variables. For HBYSAS, *U* = 10.0, *p* = 0.13; for WS, *U* = 11.0, *p* = 0.16; and for DAAS-21, *U* = 21.5, *p* = 0.82.

Research questions were analysed by conducting two mixed-design 4 by 2 [time of measurement by CSB affiliation] ANOVAs, with the times of measurement as the within-subject factor in each ANOVA. The dependent variable in each analysis was either the DASS-21 or the WSQ score. The data were analysed using IBM SPSS Statistics (Version 29). Additionally, for each effect, *post-hoc* power analyses were computed using G*Power software, version 3.1 (59).

### Results

The aim of the third research was to examine the effect of sexual abstinence on withdrawal symptoms between the two different research groups – those who affiliate with CSB and those who do not. The symptoms were measured by both the DASS-21 and WSQ in order to validate the latter. The research question was examined using two mixed-design 4 by 2 [time of measurement by CSB affiliation] ANOVAs, with the times of measurement as the within-subject factor in each ANOVA.

The first ANOVA was conducted with the DASS-21 score as the dependent variable. Mauchly’s test of sphericity was non-significant, *Mauchly’s W* = .78, χ^2^ (5) = 6.72, *p* = .24, with a power value of.98. A significant main effect for CSB affiliation was found, *F*(1, 28) = 21.38, *p* <.001, η^2^ *_p_* = .20, meaning that participants CSB group reported averaged DASS-21 scores which were higher than those reported by those without CSB. Means and standard deviations for this effect are shown in **Table 2**.

**Table 2:** Means and standard deviations of DASS-21 and WSQ scores for research groups by time of measurement.

Time of Measurement	CSBD (n = 14)		Non-CSBD (n = 16)		Total (N = 30)	
DASS-21						
	M	SD	M	SD	M	SD
Baseline	56.00	9.05	34.56	7.09	44.57	13.45
3rd day	43.79	4.77	34.00	5.49	38.57	7.10
7th day	27.64	6.49	30.88	6.12	29.37	6.40
10th day	28.36	5.26	33.31	7.18	31.00	6.74
Total	38.95	2.54	33.19	4.00		
WSQ						
	M	SD	M	SD	M	SD
Baseline	63.86	8.62	40.81	11.87	51.57	15.59
3rd day	54.36	3.10	37.63	9.35	45.43	11.03
7th day	41.21	10.46	34.44	9.17	37.60	10.22
10th day	33.43	7.05	40.00	8.66	36.93	8.50
Total	48.21	3.72	38.22	6.72		

DASS-21, the short depression, anxiety and stress scales; WSQ, withdrawal symptoms questionnaire; CSBD, Compulsive sexual behavior disorder.

A main effect for time of measurement was found: *F* (3, 84) = 96.69, *p* <.001, η^2^ *_p_* = .59, with a power value of 1. And finally, a significant time of measurement by CSB affiliation interaction was found as well, *F* (3, 84) = 27.26, *p* <.001, η^2^ *_p_* = .49, with a power value of 1. *Post-hoc* analyses of the simple effects for the time effect were conducted, using *t* tests for paired samples with Bonferroni correction (α = .05/6). Analyses revealed that the average DASS-21 score during the baseline measurement was significantly higher than the scores measured during the rest of the measurement times, with *t*(1, 29) = 3.24, *p* <.01, *Cohen’s d* = .59 for the comparison between the baseline measurement scores and the second measurement time (the 3rd day); *t*(1, 29) = 5.01, *p* <.001, *Cohen’s d* = .92 for the comparison between the baseline measurement scores and the third measurement time (the 7th day); and *t*(1, 29) = 4.46, *p* <.001, *Cohen’s d* = .81 for the comparison between the baseline measurement scores and fourth measurement time (the 10th day). The average DASS-21 score during the second measurement time was significantly higher than both the scores obtained during the third and fourth measurement times, with *t*(1, 29) = 4.62, *p* <.001, *Cohen’s d* = .84 for its comparison with the third measurement time, and *t*(1, 29) = 3.78, *p* = .001, *Cohen’s d* = .69 for its comparison with the fourth measurement time. No significant difference in the average DASS-21 score was found between the third and fourth measurement times, *t* (1, 29) = -1.15, *p* = .29, *Cohen’s d* = -.21. Means and standard deviations for this effect are shown in **Table 2**.

**Figure 5:**
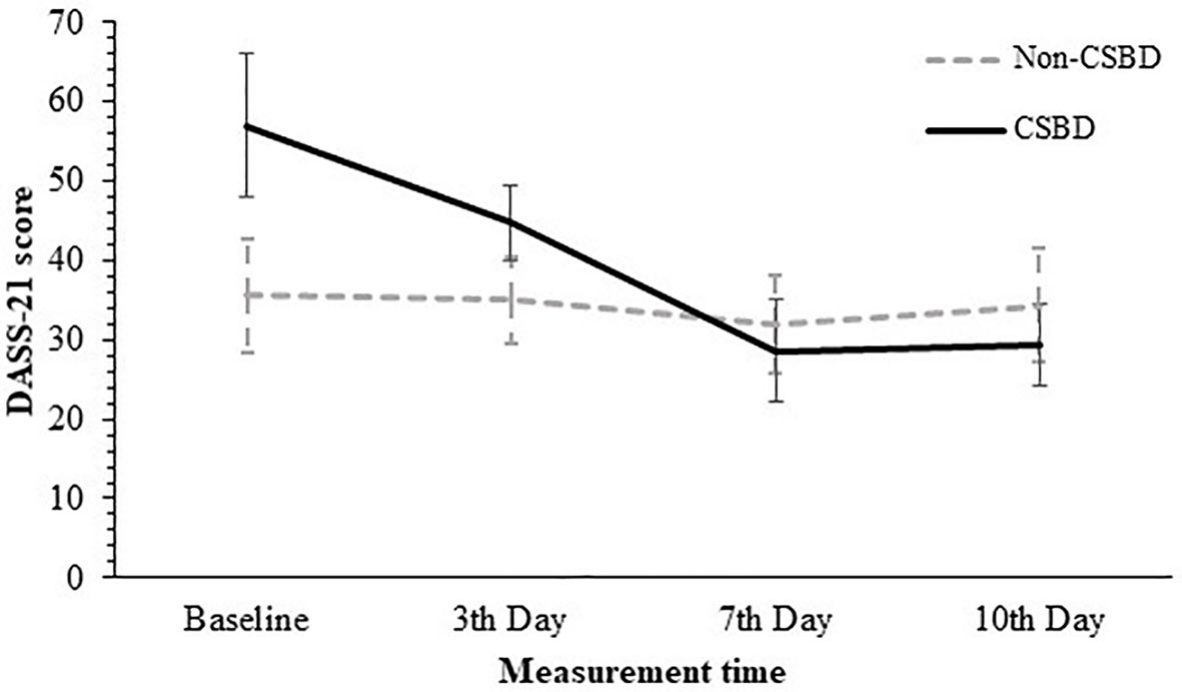
Average DASS-21 Scores by CSBD diagnosis across the Four Measurement Times. DASS-21, the short depression, anxiety and stress scales; CSBD, compulsive sexual behavior disorder (error bars show standard deviations).

*Post-hoc* analyses of the simple effects for the interaction effect were conducted, using *t* tests for paired samples with Bonferroni correction (α = .05/12). For non-CSB group all comparisons between the different times were non-significant. Between baseline and the 3^rd^, 7^th^ and 10^th^ measurements (*t* (1, 15) = 0.31, *p* = .758, *Cohen’s d* = .08; *t* (15) = 1.60, *p* = .131 *Cohen’s d* = .40; *t* (1, 15) = 0.61, *p* = .551 with *Cohen’s d* = .15, accordingly), between the 3^rd^ day, the 7^th^, and the 10^th^ (*t* (1, 15) = 1.60, *p* =. 131. *Cohen’s d* = .40; *t* (1, 15) = 0.36, *p* = .726, *Cohen’s d* = .09 accordingly), and between the 7^th^ day and the 10^th^ day (*t* (1, 15) = -0.99, *p* = .336, *Cohen’s d* = -.25.), For the CSB group all comparisons between different times were significant excluding the comparison between 7^th^ day and 10^th^ day where the comparison was non-significant. Thus, the significant comparisons were between baseline and the 3^rd^, 7^th^ and 10^th^ measurements (*t* (1, 13) = 4.77, *p* <.001, *Cohen’s d* = 1.27; *t* (1, 13) = 8.19, *p* <.0 01. *Cohen’s d* = 2.19.; *t* (1, 13) = 8.267, *p* <.001 with *Cohen’s d* = 2.32, accordingly), between the 3^rd^ day, the 7^th^, and the 10^th^ day (*t* (1, 13) = 6.13, *p* <. 001 *Cohen’s d* = 1.64; *t* (1, 13) = 6.69, *p* <.001 *Cohen’s d* = 1.79) and between 7^th^ day and 10^th^ day (*t* (1, 13) = -0.58, *p* = -.16 *Cohen’s d* = .93). Means and standard deviations detailed in **Table 2** above. The results are illustrated in **Figure 5**.

Similar pattern of results emerged from the ANOVA in which the WSQ score as the dependent variable. The WSQ score was calculated as the sum of the individual item scores.Specifically, *Mauchly’s W* = .75, χ^2^ (5) = 7.55, *p* = .18. A significant main effect for CSB affiliation was found, *F* (1, 28) = 24.35, *p* <.001, η^2^ *_p_* = .47, with a power value of 1, meaning that participants with CSB reported averaged WSQ scores that were higher than those reported by those without CSB. Means and standard deviations for this effect are shown above in **Table 2**.A main effect for time of measurement was found: *F* (3, 84) = 24.48, *p* <.001, η^2^ *_p_* = .47, with a power value of 1. Finally, a significant time of measurement by CSB affiliation interaction was found: *F* (3, 84) = 18.88, *p* <.001, η^2^ *_p_* = .40, with a power value of 1. *Post-hoc* analyses of the simple effects for the time effect were conducted, using *t* tests for paired samples with Bonferroni correction (α = .05/6). The analyses revealed that the average WSQ score during the baseline measurement was higher than the scores measured during the rest of the measurement times. Specifically, the WSQ average scores at the baseline measurement were higher than the score at the second measurement time (the 3rd day), *t* (1, 29) = 3.46, *p* <.01, *Cohen’s d* = .63. The WSQ average scores at the baseline measurement were higher than the scores measured at the third measurement time (the 7th day), *t* (1, 29) = 4.83, *p* <.001, *Cohen’s d* = .88; and at the fourth measurement time (the 10th day), *t* (1, 29) = 4.10, *p* <.001, *Cohen’s d* = .81. The average WSQ score during the second measurement time was higher than both the scores obtained during the third and fourth measurement times, with significance rates of *t*(1, 29) = 3.64, *p* <.001, *Cohen’s d* = .66 and *t*(1, 29) = 2.93, *p* <.01, *Cohen’s d* = .54 accordingly. No significant difference in the average WSQ score was found between the third and fourth measurement times, *t* (1, 29) = 0.29, *p* = .78, *Cohen’s d* = .05. Means and standard deviations for this effect are shown in **Table 2**.

*Post-hoc* analyses of the simple effects for the interaction effect were conducted, using *t* tests for paired samples with Bonferroni correction (α = .05/12). The results show that for non-CSB group all comparisons between the different times were non-significant. Between baseline and the 3^rd^, 7^th^ and 10^th^ measurements (*t*(1, 15) = 1.20, *p* = .249, *Cohen’s d* = .30; *t*(1, 15) = 2.01, *p* = .063 *Cohen’s d* = .50; *t*(1, 15) = 0.28, *p* = .786 with *Cohen’s d* = .07, accordingly), between the 3^rd^ day, the 7^th^, and the 10^th^ day (*t*(1, 15) = 1.24, *p* = .234 *Cohen’s d* = .31; *t*(1, 15) = -0.78, *p* = .447 *Cohen’s d* = -.20) and between 7^th^ day and 10^th^ day (*t*(1, 15) = -1.75, *p* = .100 *Cohen’s d* = -.44). For the CSB group all comparisons between different times were significant excluding the comparison between 7^th^ day and 10^th^ day where the comparison was non-significant. Thus, the significant comparisons were between baseline and the 3^rd^, 7^th^ and 10^th^ measurements (*t* (1, 13) = 4.70, *p* <.001, *Cohen’s d* = 1.26; *t* (1, 13) = 5.69., *p* <.0 01. *Cohen’s d* = 1.52.; *t*(1, 13) = 8.23, *p* <.001 with *Cohen’s d* = 2.20., accordingly), between the 3^rd^ day, the 7^th^, and the 10^th^ day (*t*(1, 13) = 4.29., *p* =. 001 *Cohen’s d* = 1.15; *t*(1, 13) = 8.81, *p* <.001 *Cohen’s d* = 2.35.) and between 7^th^ day and 10^th^ day (*t*(1, 13) = 3.47, *p* <.01 *Cohen’s d* = .93).

**Figure 6:**
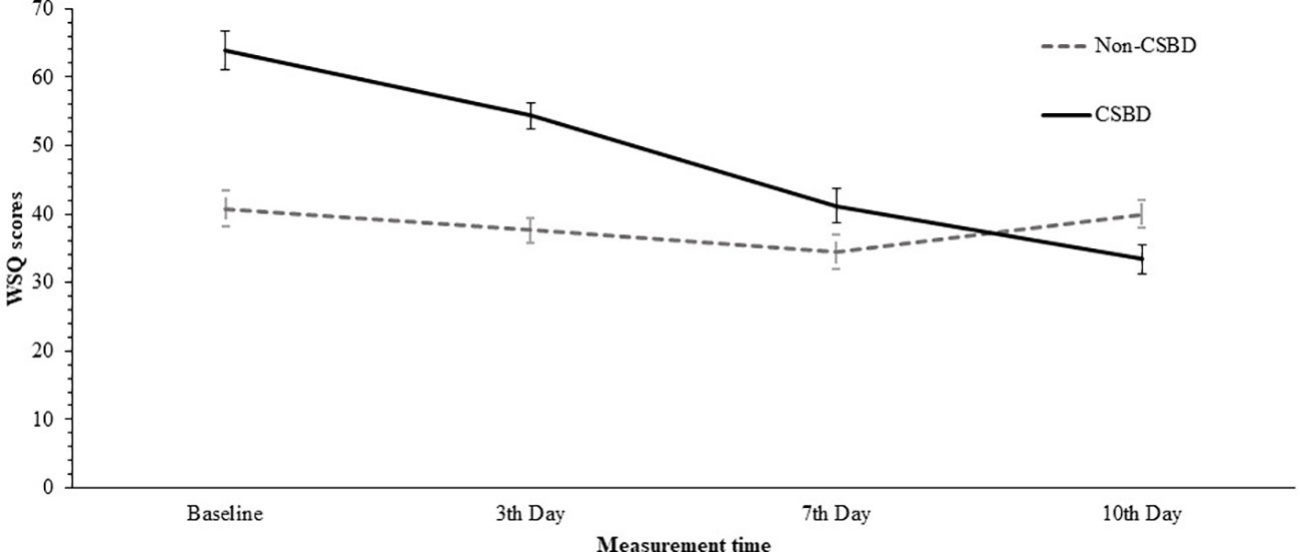
Average WSQ Scores by Research Groups across Measurement Times. WSQ, Withdrawal symptoms questionnaire; CSBD, compulsive sexual behavior disorder (error bars show standard deviations).

The means and standard deviations are detailed in **Table 2**. The descriptive statistics are detailed in **Table 2**. **Figure 6** shows Average WSQ scores by CSB affiliation across the four measurement times.

### Discussion

This study found that individuals with CSB experienced improvements in various symptoms after abstaining from sexual activity. That was evident as early as the 3^rd^ day after abstention. This improvement persisted from the 3^rd^ day to the 7th day and remained consistent in the assessment on the 10^th^ day. As previously noted, withdrawal symptoms typically emerge in the initial days of abstention, with subsequent relief over time. The results of this study suggest that refraining from sexual activity, among individuals with CSB, led to improvements in withdrawal symptoms as expected. It is worth emphasizing that at baseline prior to any abstinence, CSB individuals reported more “withdrawal symptoms” compared to non-CSB individuals. However, as the days of abstinence progressed, the consistent reduction in “withdrawal symptoms” led the CSB group to eventually reach to the same levels of symptoms reported by non-CSB individuals.

There are conflicting findings of withdrawal symptoms in previous studies in CSB. Fernandez et al. (86), found no correlation between problematic pornography use and withdrawal symptoms. However, they observed that individuals reporting both high levels of problematic pornography use and additionally reporting of 4 weeks of daily engagement with pornography, experienced heightened cravings during periods of abstinence. The authors offer two potential explanations for this phenomenon. The first explanation proposes that the heightened cravings indicate withdrawal symptoms, providing evidence of addiction within this group. The second explanation, according to the authors, suggests that the absence of other withdrawal symptoms, coupled with increased desire levels, indicates non-pathologically elevated sexual desire in this group. Lewczuk et al. (74) encountered a similar dilemma regarding high sexual desire during periods of sexual abstinence. Their study revealed that participants, upon retrospective self-reporting, experienced withdrawal symptoms primarily characterized by increased desire and arousal when attempting to curb or abstain from sexual habits. The authors suggested that this heightened desire may stem from factors unrelated to withdrawal itself, proposing the possibility of attributing it to preliminary factors. Furthermore, they suggested that unfulfilled desire during abstinence could lead to concentration difficulties, feelings of inadequacy, frustration, and mood disturbances in some individuals, while others may benefit from abstaining or reducing sexual activity by freeing time for engagement in alternative positive activities. Similarly, Fernandez et al. (87) broadly reviewed various possible addictive behaviours, such as gaming, running, and pornography watching. Their findings on pornography addiction underscore its positive impact on interpersonal functioning and improved self-regulation.

An inherent limitation of our study is that participants voluntarily chose or believed they could abstain from sexual activity, despite having a history of excessive sexual behaviour. Further research is needed to explore whether differing levels of readiness for abstaining from sexual activity influence the development of withdrawal symptoms. Another limitation of the current study is the inability to guarantee that participants were actually sexually abstinent. While participants were instructed to abstain from sexual activity, compliance was based on self-reporting, which may introduce potential bias in the results.

## General discussion

Compulsive Sexual Behaviour Disorder (CSBD), akin to other impulse control and addictive disorders, prompts inquiry into the underlying mechanisms driving such behaviour despite apparent negative consequences for the individual’s well-being. Since the term CSBD is based on the ICD-11 definition, and in the current research we relied on Griffiths (22) addiction framework as reflected in the HBYSAS questionnaire, we used the term CSB to emphasize this distinction. Our research revealed that a negative mood, or the absence of a positive mood—specifically, a state of depression—significantly reduced the inclination for pornography consumption among all participants, including those with CSB. Considering one theoretical perspective that views CSB as a compulsive disorder, it’s noteworthy that depression correlates positively with obsession symptoms but lacks any association with compulsive behaviours, as indicated by Ricciardi and McNally (88). Similarly, OCD symptoms were found to correlate with Negative Affect (NA) but not with Positive Affect (PA), as noted by Fergus and Wu (89). The absence of a relationship between PA and compulsive behaviours implies that depression neither counters nor diminishes the urge to engage in compulsive acts. Therefore, our findings of diminished pornography craving during a depressed mood state, suggest that CSB as manifested in our sample isn’t inherently compulsive. Additionally, we observed that compared to the non-CSB group, individuals with CSB reported higher levels of wanting to watch pornography across all stages of exposure to sexual stimulation, but not higher levels of ‘liking’. However, their wanting was not increased as a function of explicitness. Both groups experienced an increase in wanting and liking as function of increased explicitness. In the third study, withdrawal symptoms have reduced during abstinence from sexual activity for CSB.

In the early review by Kor et al. (90) the authors have discussed the similarities and differences between hypersexual disorder, drug addiction, and pathological gambling. They have concluded that despite many similarities between the features of hypersexual behaviour and substance-related disorders, the research on hyper sexuality disorder at this time is in its infancy and much remains to be learned before definitively characterizing hyper sexuality disorder as an addiction at this time. Later on, Kraus et al. (20) have reviewed the evidence for regarding CSB as a behavioural addiction from epidemiological, phenomenological, clinical and biological domains with respect to data from substance and gambling addictions. They have found overlapping features between CSB and substance-use disorders such as common neurotransmitter systems and similar craving and attention biases that were shown by recent neuroimaging studies. Also, similar pharmacological and psychotherapeutic treatments may be applicable to CSB and substance addictions, although there is little evidence at the moment to support that. The authors have concluded that despite the growing body of research linking compulsive sexual behaviour to substance addictions, significant gaps in understanding continue to complicate the classification of compulsive sexual behaviour as an addiction.

In the recent past, we arrived at a similar conclusion to the reviews mentioned regarding the insufficient information to resolve the classification issue (13). The current research results add complexity to the challenge of identifying a category of Axis I disorders that will comprehensively capture and reflect the phenomenology of CSB. Our three studies seem to present conflicting support for the behavioural model of CSB. Although there is some evidence for incentive sensitization theory indicated by wanting to watch pornography, it does not grow as function of explicitness level. There is also evidence for a reduction of withdrawal symptoms during abstinence but no evidence that mood induction techniques increase craving. In fact, negative mood can actually reduce craving.

Furthermore, the findings that both groups experienced a reduction of craving following negative mood induction also supports the argument that CSB is not distinctly different from normal sexual behaviour. As mentioned earlier, Fernandez et al. (86) were also inclined to interpret their findings by asserting that heightened sexual desire, even in the context of CSB, may not necessarily be inherently pathological. Under certain circumstances, what we refer to as “compulsive sexuality” could be described as an expression of desire. Some individuals adopt a lifestyle akin to sexual addiction, excluding the element of distress, and therefore, they do not seek treatment (91). Nevertheless, the excessive sexual activity causes conflict and distress among those suffering from CSB and an explanation is required for the mechanism that preserves and encourages this behaviour. As Gold and Heffner (92) suggested, each one of the models of CSB accounts for a subgroup of cases rather than for the whole phenomena. Any overriding definition lacks clinically empirical research and we need to avoid talking about CSB as a homogeneous group and remember that under the title of CSB there are several forms of pathology that may correspond with different structures of mental disorders (93).

Our findings may contribute to the understanding of CSB as an axis 2 disorder as discussed by Montaldi (94). Specifically, while excessive sexual behaviour is maladaptive, it should not be regarded as inherently pathological. Instead, it represents a turbulent and maladaptive personality style that manifests through sexual gratification. It is important to differentiate between individuals with CSB, who experience significant distress and impairment due to their symptoms, and those with high sexual motivation without such impairment. The latter should not be classified as having CSB, as the diagnosis is defined by the presence of suffering and impairment resulting from the symptoms. A similar claim can be found in the chapter we recently published (95) in which we claimed that CSB can be an ego syntonic action that is exerted with a sense of ‘urgency’. Whiteside and Lynam (96) describe urgency in this context as a personality style that tends to experience emotions in a stormy manner and carry them out with a sense of necessity. ‘Urgency’ as a trait or temporal affective state can carry either negative emotion like anger or positive emotion like enthusiasm. Accordingly, a maladaptive course of behaviour can be the result of either positive urgency or negative urgency. As part of this claim, it is important for us to denote the difference between acting out ‘negative-urgency’ ‘mood-modification’ as tested in this study, which is a criterion in addiction disorder. While in mood modification, the behaviour in question serves as escapism and distraction, acting out of negative urgency might be interpreted as an expression of inner turmoil, which can be seen as ego-syntactic or ego-consistent (94). In other words, it can be said that in addiction or compulsive acts, the individual seeks to avoid an inner experience through the addictive or compulsive behaviour. On the other hand, in hyper sexuality, at least one that is characterized by a sense of urgency, the individual seeks to experience the situation fully and excessively. A sense of urgency, either negative or positive, requires a sense of energy and vitality that is absent during a depressed mood state, as shown in our study following the negative movie.

It can be argued that in gambling disorder or excessive shopping, the thrill from gambling or unreasonable shopping is not pro-adaptive in any context. On the other hand, sexual pleasure, when it keeps its healthy quality, should not be seen as a pathological act, regardless of its frequency. Under this perception, there is an answer to the voices criticizing the very idea of pathologizing non-paraphilic sexual behaviour solely because of the frequency with which it is practiced. Classification as a mental disorder is required only because the individual encounters difficulty maintaining a balance between the demands of an adaptive lifestyle and the way in which he finds a way to express a strong emotional drive.

A deeper understanding of the emotional experience accompanying situations of conflictual heightened sexuality can contribute both to diagnosis and treatment. These studies can instil hope in patients by portraying the connection between mood and sexuality, emphasizing that high libido is not inherently pathological in itself. Furthermore, our observation of the absence of withdrawal symptoms aligns with other research, reinforcing the encouragement for treatment candidates to adhere to the process. As described by Fernandez et al. (87), abstinence proves rewarding when sustained. Successfully attaining and maintaining abstinence is acknowledged to be quite challenging due to ingrained behavioural patterns. However, the multitude of positive effects reported by individuals practicing abstinence suggests that refraining from pornography could potentially serve as a beneficial intervention for conditions such as CSB.

When considering the limitations of this research, it is important to note that all three studies relied on small and convenience-based samples. In addition to the limitations specific to each study, these sampling constraints collectively impact the generalizability of the findings. Furthermore, the populations in the first and third studies likely consisted of individuals who were relatively motivated and mentally resilient. These factors should prompt caution in interpreting the results, particularly regarding conclusions that might question the conceptualization of CSB as a behavioural addiction. At the same time, the findings from the current studies show at least the existence of a subgroup for whom sexual activity is associated with distress but does not have pathological qualities apart from the toll it takes on personal functioning and the emotional states it indicates. This finding has significant potential implications for treatment. While for addiction disorders or OCD, the standard treatment aims to extend the ability to bear the urge without actualizing it, in cases of CSB such as we propose here, the treatment should focus on the personality patterns and the emotional meanings that sex has for the individual.

In summary, the aim of the current article was to examine, through three studies, whether CSB fits with the addiction model. The first experiment investigated the influence of different mood states on the desire to watch pornography. The second study examined whether CSB individuals experience sensitization or tolerance in response to pornography viewing. The third study focused on the impact of abstaining from sexual activity on withdrawal symptoms. We discovered conflicting evidence in support of CSB as a behavioural addiction. In opposite to what we predicted we found no evidence that mood induction increases craving for pornography. Furthermore, both the CSB and non-CSB exhibited reduced pornography craving following exposure to the negative mood movie. Moreover, CSB individuals experienced both liking and wanting over increased levels of sexual explicitness. Like a previous study (41) they showed increased wanting and reduced liking in accordance with Incentive Sensitization theory would predict. Lastly, we observed that abstaining from sex reduced withdrawal symptoms for individuals with CSB, like previous evidence of withdrawal from substances of abuse.

## Data Availability

The raw data supporting the conclusions of this article will be made available by the authors, without undue reservation.
